# Methyl-CpG-binding protein 2 drives the Furin/TGF-β1/Smad axis to promote epithelial–mesenchymal transition in pancreatic cancer cells

**DOI:** 10.1038/s41389-020-00258-y

**Published:** 2020-08-26

**Authors:** Huizhi Wang, Jie Li, Junbo He, Yawen Liu, Wen Feng, Hailang Zhou, Meng Zhou, Hong Wei, Ying Lu, Wanxin Peng, Fengyi Du, Aihua Gong, Min Xu

**Affiliations:** 1 Department of Gastroenterology, Affiliated Hospital of Jiangsu University, Jiangsu University, 438 Jiefang Road, Zhenjiang, 212000 China; 2 Department of Gastroenterology, The First People’s Hospital of Jingzhou, 8 Aviation Road, Jingzhou, 434000 China; 3 Department of Cell Biology, School of Medicine, Jiangsu University, 301 Xuefu Road, Zhenjiang, 212000 China

**Keywords:** Cell growth, Cell migration, Pancreatic cancer

## Abstract

Methyl-CpG-binding protein 2 (MeCP2) has been characterized as an oncogene in several types of cancer. However, its precise role in pancreatic ductal adenocarcinoma (PDAC) remains unclear. Hence, this study aimed to evaluate the potential role of MeCP2 in pancreatic cancer progression. We found that MeCP2 was upregulated in pancreatic cancer tissues, enhanced migration, invasion, and proliferation in pancreatic cancer cells, and promoted tumorigenesis. Further evidence revealed that MeCP2 remarkably increased the mesenchymal markers vimentin, N-cadherin, and Snail, and downregulated the expression of the epithelial markers E-cadherin and ZO-1, indicating that MeCP2 promotes epithelial–mesenchymal transition (EMT). In addition, we found that MeCP2 upregulated the expression of Furin, activated TGF-β1, and increased the levels of p-Smad2/3. Importantly, we demonstrated that MeCP2, as a coactivator, enhanced Smad3 binding to the *furin* promoter to improve its transcription. Therefore, MeCP2/Smads drive the expression of Furin to activate TGF-β1, and in turn, phosphorylate Smad2/3, which forms a positive-feedback axis to promote EMT in pancreatic cancer cells.
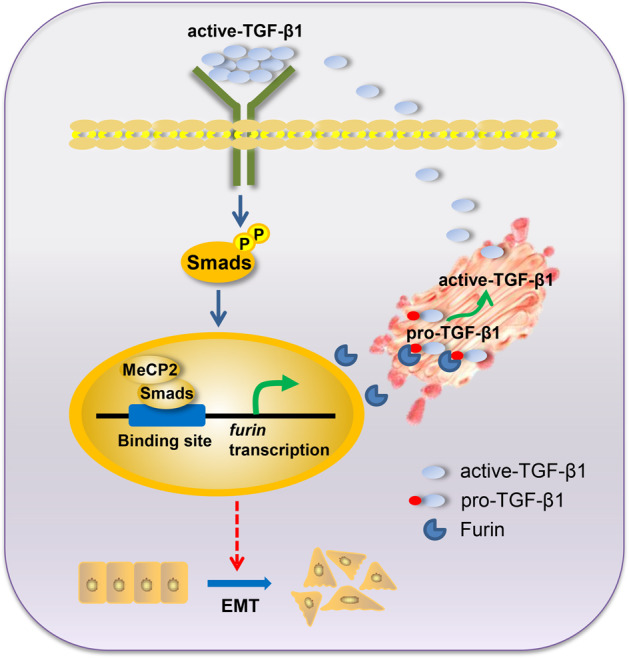

## Introduction

Pancreatic ductal adenocarcinoma (PDAC), the most common type of pancreatic cancer, is an extremely lethal disease characterized by mortality closely reflecting incidence. Because of the absence of reliable early biomarkers and the lack of efficient therapies, the 5-year survival rate remains below 9%^1^. Despite decades of effort, the underlying mechanisms of pancreatic cancer remain far from fully understood. Thus, there is an urgent need to discover the mechanism of pancreatic cancer to facilitate the prediction of cancer risk and diagnose this disease at an earlier stage.

Epithelial–mesenchymal transition (EMT) is a critical step at several stages during tumor progression, that is characterized by a breakdown of cell junctions and the loss of epithelial characteristics and cell polarity, leading to cancer progression^2^. Moreover, it has been reported that EMT plays a significant role in the migration and invasion of various malignant cells, including pancreatic cancer cells. Meanwhile, it is generally accepted that the activation of TGF-β1/Smad signaling is critical to induce EMT^3,4^. Furin has been reported to promote the migration and invasion abilities of human cancer cells via pro-MT1-MMP and/or pro-TGF-β1 activation^5,6^. Furin is a member of the family of subtilisin/kexin-like proprotein convertases (PCs), which contribute to the activation of precursor pro-proteins to become biologically active^7^.

Methyl-CpG-binding protein 2 (MeCP2) is a key member of the methyl-CpG-binding domain proteins (MBDs), which are important components of the DNA methylation machinery^8^. The classical model of MeCP2 function delineates its role in gene suppression through binding to methylated CpG dinucleotides in promoters. Nevertheless, recent discoveries have proven that MeCP2 is a multifunctional protein involved not only in transcriptional silencing but also in transcriptional activation, chromatin compaction, and modulation of RNA splicing^9^. The hypothesis has especially been focused on MeCP2 acting as a transcriptional activator to stimulate gene expression, which has been confirmed in the hypothalamus^10^. Recently, it has been found that MeCP2 is involved in the development of tumors in different cancers, such as gastric carcinoma^11^, cervical cancer^12^, and prostate cancer^13^. However, the role of MeCP2 in pancreatic cancer remains to be clarified.

In this study, we demonstrated that MeCP2 was markedly different in pancreatic cancer tissues, enhanced the proliferation, migration, and invasion abilities of pancreatic cancer cells, and promoted tumorigenesis. Furthermore, it was found that MeCP2, as a coactivator, enhanced Smad3 binding to the *furin* promoter to activate Furin/ TGF-β1/Smad signaling resulting in the promotion of EMT in pancreatic cancer cells. All these findings prove for the first time that MeCP2 might be a promoter in pancreatic cancer progression.

## Results

### MeCP2 is profiled in pancreatic cancers and different pancreatic cancer cells

**Fig. 1:**
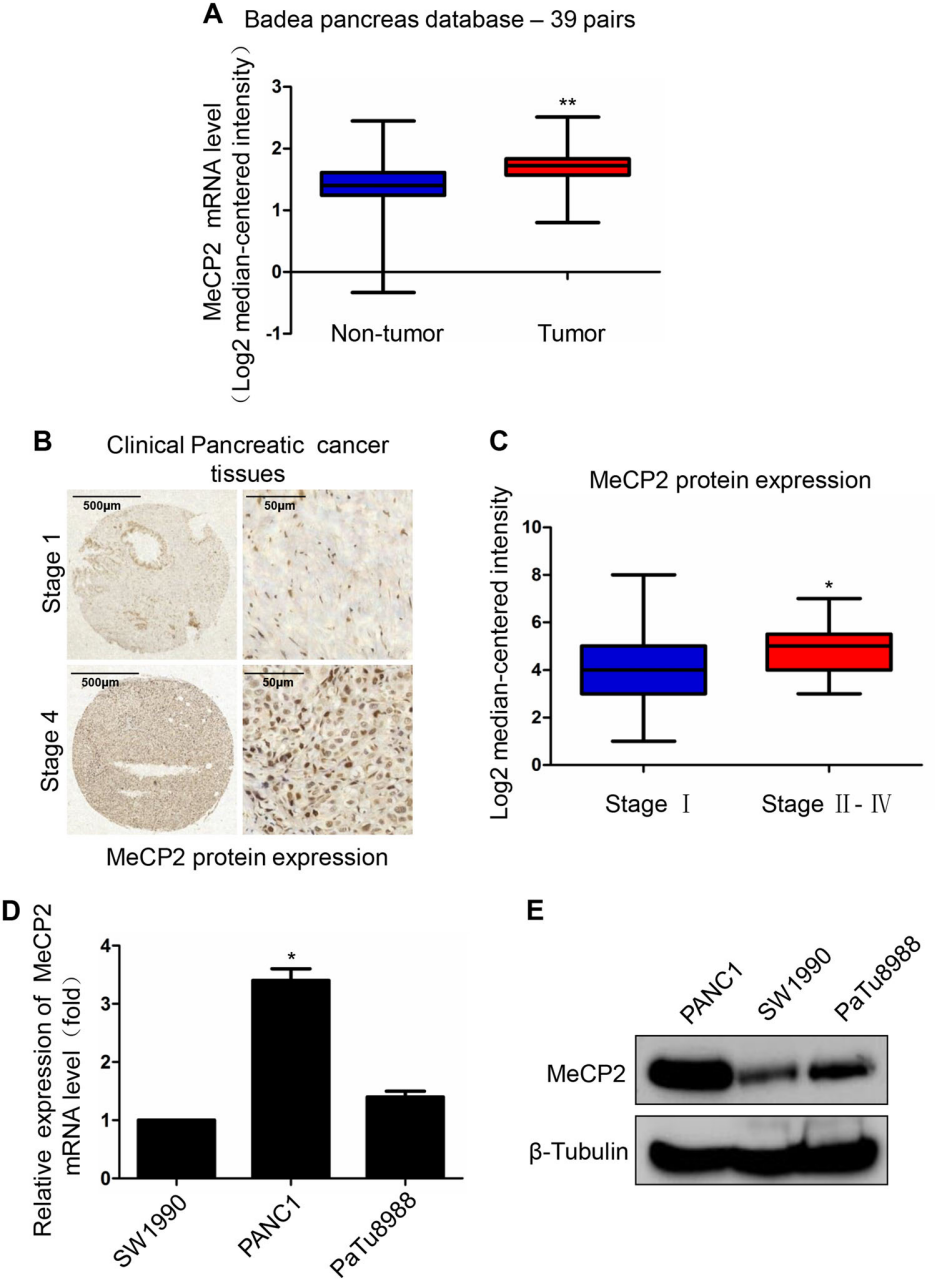
Methyl-CpG-binding protein 2 (MeCP2) is profiled in pancreatic cancers and different pancreatic cancer cells. **a** Analysis of MeCP2 mRNA levels in 78 pairs of pancreatic cancer and nontumor tissues in Badea pancreas database. *N* = 39 for nontumor group, and *N* = 39 for tumor group. **P < 0.01. **b**, **c** Analysis of the MeCP2 protein level indicates MeCP2 correlates with the stage. *N* = 42 for stage I group, *N* = 33 for stage II–IV group. *P < 0.05. **d** Relative expression levels of MeCP2 mRNA were assessed in SW1990, PaTu8988, and PANC1 cells. *P < 0.05. **e** Relative expression levels of MeCP2 protein were assessed in SW1990, PaTu8988, and PANC1 cells.

To confirm the clinical relevance of MeCP2 expression, we first analyzed MeCP2 mRNA expression in the Badea pancreas database. We found that the MeCP2 mRNA level was higher in pancreatic cancer tissues than in normal pancreatic tissues (1.724 ± 0.05294 vs. 1.431 ± 0.07816, *P* < 0.01, *n* = 78) (Fig. 1a). To evaluate the correlation of MeCP2 expression and clinicopathological features, we further interrogated the TCGA database (https://genome-cancer.ucsc.edu). We analyzed the mRNA level of MeCP2 at different stages and found that MeCP2 expression was higher in stage II–IV groups than in the stage I group (Fig. 1b, c). Next, we assessed the expression of MeCP2 in three pancreatic cancer cell lines, PANC1, SW1990, and PaTu8988, using real-time PCR and western blotting. The results showed that MeCP2 mRNA and protein levels were higher in PANC1 cells than in SW1990 and PaTu8988 cells (Fig. 1d, e). In addition, we found that PANC1 cells had poorer differentiation and were more invasive compared to the other two cell lines (Supplementary Fig. S1a–c), indicating that MeCP2 was associated with the state of differentiation or the malignant phenotype of these cells. Subsequently, we constructed shMeCP2 and Flag-MeCP2 plasmids to investigate the roles of MeCP2 in pancreatic cancer, with shEGFP or vector as a control, respectively. In previous study, we found that MeCP2 expression, at both the mRNA and protein levels, was highest in PANC1 cells, intermediate in PaTu8988 cells, and lowest in SW1990 cells. Therefore, we used PANC1 and PaTu8988 cells with higher MeCP2 expression in shRNA experiments and SW1990 and PaTu8988 cells with relatively lower MeCP2 expression in MeCP2 overexpression assays. After infection, the mRNA and protein levels of MeCP2 were significantly reduced in the shMeCP2 group compared with the shEGFP group (Supplementary Fig. S1d, e). Vector or Flag-MeCP2 was transfected into PaTu8988 and SW1990 cells, and MeCP2 overexpression was confirmed at the mRNA and protein levels by real-time PCR and western blotting (Supplementary Fig. S1f, g).

### MeCP2 promotes proliferation of pancreatic cancer cells and tumorigenesis

**Fig. 2:**
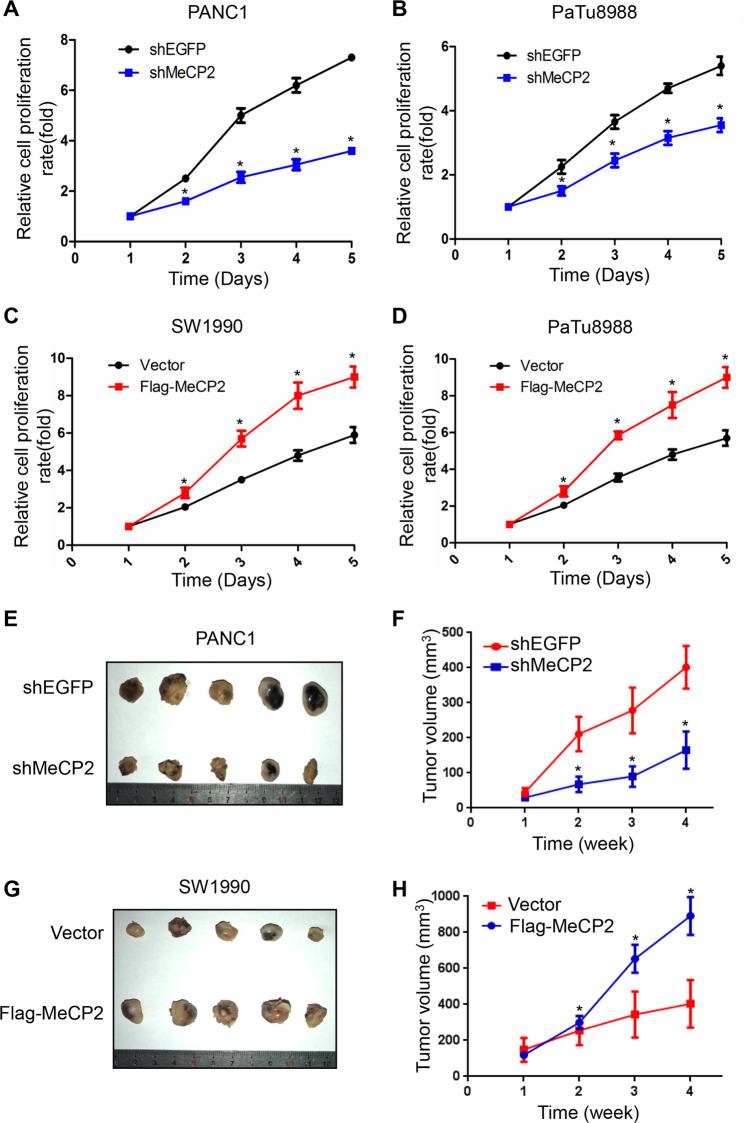
Methyl-CpG-binding protein 2 (MeCP2) promotes proliferation of pancreatic cancer cells and tumorigenesis. **a** MeCP2 knockdown resulted in a lower proliferation of PANC1 cells at 2, 3, 4, and 5 days after transfection. **b** MeCP2 knockdown resulted in a lower proliferation of PaTu8988 cells at 2, 3, 4, and 5 days after transfection. **c** At 1, 2, 3, 4, and 5 days after transfection with Flag-MeCP2, SW1990 cell proliferation was determined by CCK8 assays. **d** At 1, 2, 3, 4, and 5 days after transfection with Flag-MeCP2, PaTu8988 cell proliferation was determined by CCK8 assays. *P < 0.05. **e**–**h** PANC1 and SW1990 cells with MeCP2 downregulation and upregulation were injected (2.0 × 10^6^ cells/site) subcutaneously into mice, and the tumor volume was measured weekly (*n* = 5 mice). The tumors were collected from 4th week. *P < 0.05.

To determine the role of MeCP2 in the proliferation of pancreatic cancer cells, we used the CCK8 assay to generate growth curves. We found that MeCP2 knockdown caused a lower proliferation of PANC1 and PaTu8988 cells at 2, 3, 4, and 5 days after infection (Fig. 2a, b). Similarly, MeCP2 overexpression enhanced the cell proliferation ability at 2, 3, 4, and 5 days in SW1990 and PaTu8988 cells (Fig. 2c, d). Furthermore, experiments in the xenograft mouse model proved that MeCP2 also significantly increased tumor growth (Fig. 2e–h). These results suggest that MeCP2 is critical for cell proliferation in pancreatic cancer cells.

### MeCP2 enhances the ability of migration and invasion in pancreatic cancer cells

**Fig. 3:**
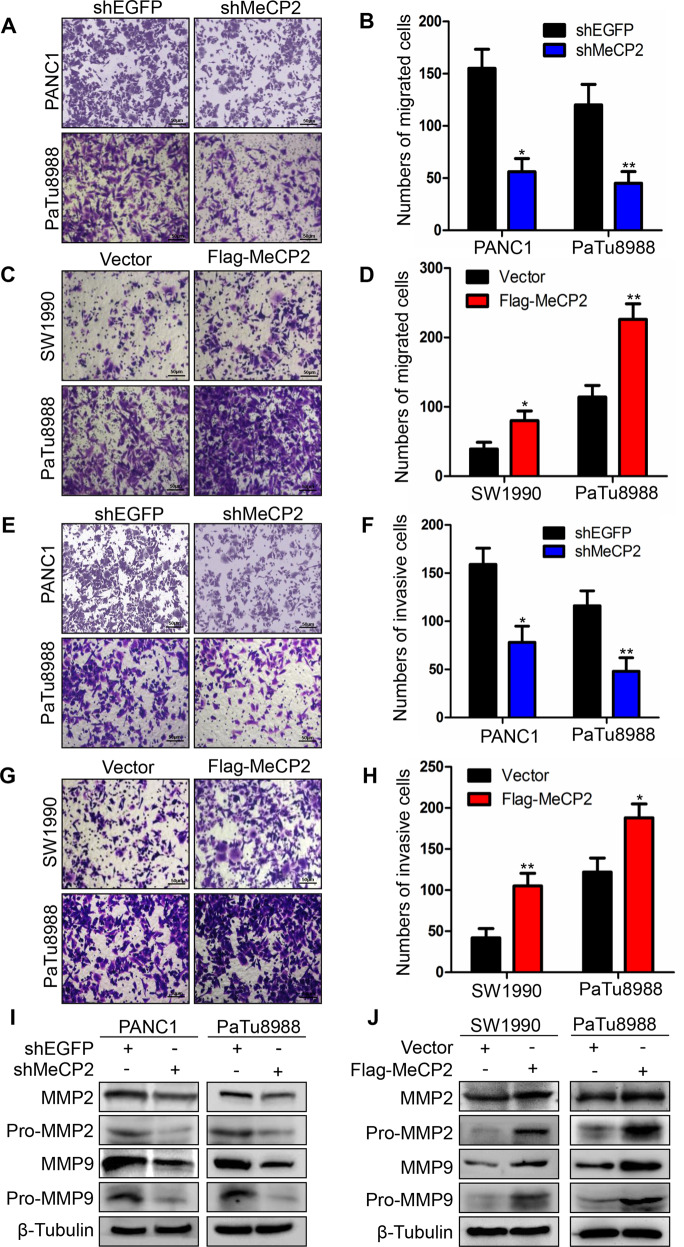
Methyl-CpG-binding protein 2 (MeCP2) enhances the migration and invasion ability of pancreatic cancer cells. **a**, **b** The ability of migration was examined using transwell assays in PANC1 and PaTu8988 cells transfected with shMeCP2 or shEGFP plasmids. Representative images of migrated cells are shown. The graph indicated the average number of migrated cells per field. *P < 0.05, **P < 0.01. **c**, **d** The ability of migration was examined using transwell assays in SW1990 and PaTu8988 cells transfected with vector or Flag-MeCP2 plasmids. Representative images of migrated cells are shown. The graph indicates the average number of migrated cells per field. *P < 0.05, **P < 0.01. **e**, **f** The invasion ability was examined using BD Matrigel invasion assays in PaTu8988 and PANC1 cells transfected with shEGFP or shMeCP2 plasmids. Invasive cells were counted and analyzed. *P < 0.05, **P < 0.01. **g**, **h** The invasion ability was examined using BD Matrigel invasion assay in PaTu8988 and SW1990 cells transfected with vector or Flag-MeCP2 plasmids. Invasive cells were counted and analyzed. **P < 0.01, *P < 0.05. **i**, **j** MMP2 and MMP9 were identified using western blotting.

Next, we examined the migration ability of pancreatic cancer cells by transwell assays. We found that the numbers of migrated cells were 154 ± 14 and 56 ± 9 (*P* < 0.05) in shEGFP and shMeCP2 PANC1 cells, and 120 ± 12 and 45 ± 8 (*P* < 0.01) in shEGFP and shMeCP2 PaTu8988 cells, respectively (Fig. 3a, b), indicating that MeCP2 knockdown significantly decreased their migration ability. To confirm the above results, we assessed the migration ability of PaTu8988 cells and SW1990 cells transfected with the vector or the Flag-MeCP2 plasmid. The numbers of migrated cells were 39 ± 6 and 80 ± 10 (*P* < 0.05) in vector and Flag-MeCP2 SW1990 cells, and 114 ± 12 and 226 ± 16 (*P* < 0.01) in vector and Flag-MeCP2 PaTu8988 cells, respectively (Fig. 3c, d), indicating that upregulation of MeCP2 significantly increased their migration ability. All these data suggest that MeCP2 enhances the migration ability of pancreatic cancer cells. Subsequently, we examined the invasion ability by BD Matrigel invasion assays. PANC1 and PaTu8988 cells were transfected with the shEGFP or shMeCP2 plasmid, and PaTu8988 and SW1990 cells were transfected with the vector or Flag-MeCP2 for 72 h. The numbers of invasive cells were 159 ± 12 and 78 ± 12 (*P* < 0.05) in shEGFP and shMeCP2 PANC1 cells, and 116 ± 11 and 48 ± 10 (*P* < 0.01) in shEGFP and shMeCP2 PaTu8988 cells, respectively (Fig. 3e, f), indicating that MeCP2 knockdown obviously suppressed the invasion ability of PANC1 and PaTu8988 cells. In addition, the numbers of invasive cells were 42 ± 8 and 105 ± 11 (*P* < 0.01) in vector and Flag-MeCP2 SW1990 cells, and 122 ± 12 and 188 ± 12 (*P* < 0.001) in vector and Flag-MeCP2 PaTu8988 cells, respectively (Fig. 3g, h), suggesting that upregulation of MeCP2 significantly enhanced the invasion ability of PaTu8988 and SW1990 cells. In addition, MeCP2 knockdown inhibited the expression of MMP2 and MMP9 (pro- and total) (Fig. 3i and Supplementary Fig. S2a, b, e, f), while MeCP2 overexpression resulted in upregulation of MMP2 and MMP9 (pro- and total) (Fig. 3j and Supplementary Fig. S2c, d, g, h). All these findings indicate that MeCP2 enhances the invasion ability of these three kinds of pancreatic cancer cells.

### MeCP2 drives TGF-β1/Smad signaling to promote EMT in pancreatic cancer cells

**Fig. 4:**
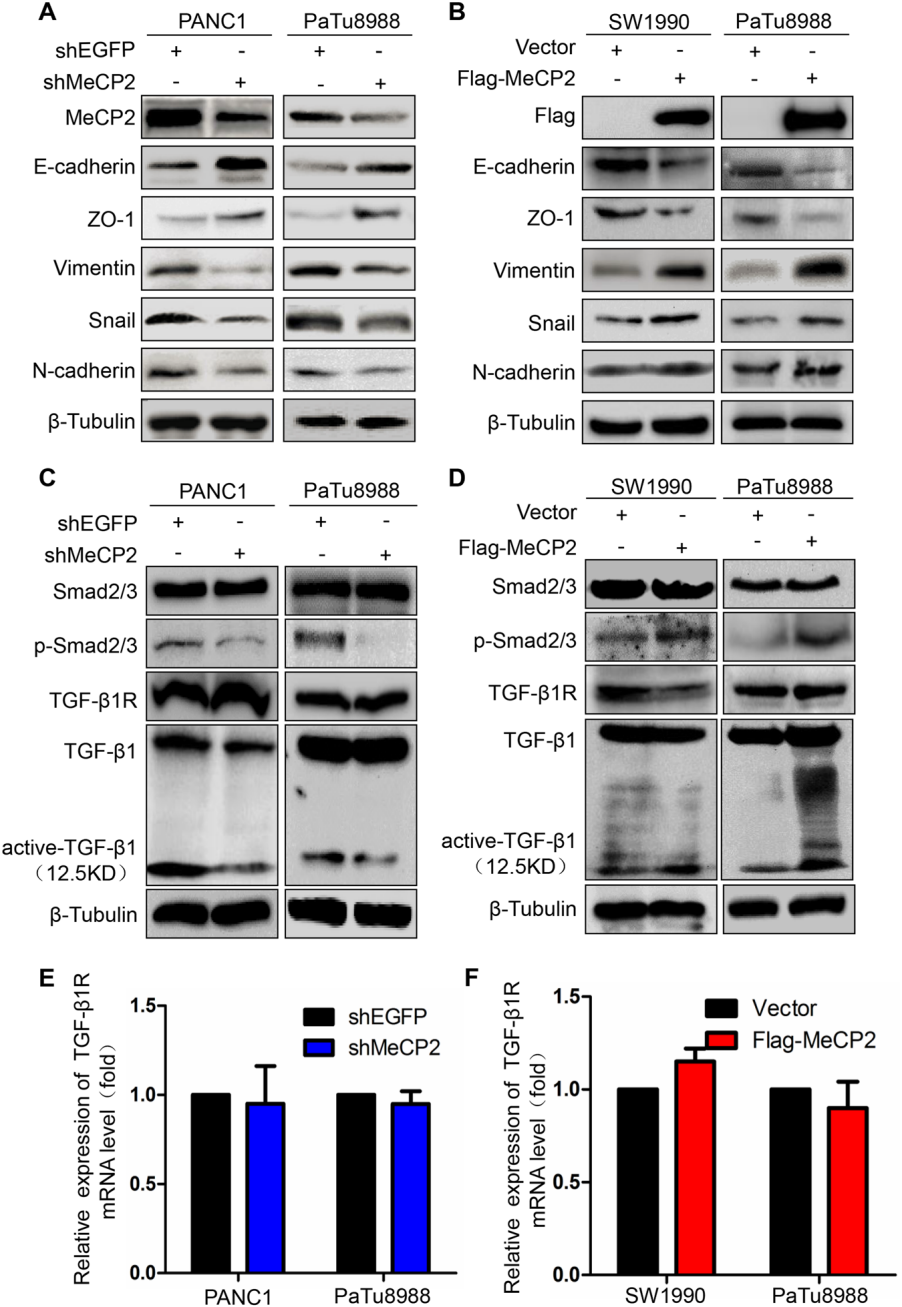
Methyl-CpG-binding protein 2 (MeCP2) drives TGF-β1/Smad signaling to promote the epithelial–mesenchymal transition (EMT) in pancreatic cancer cells. **a** Knockdown of MeCP2 in PANC1 and PaTu8988 cells reversed EMT, as detected by an increase in E-cadherin, ZO-1 and decreases in vimentin, Snail, and N-cadherin. **b** Treatment of PaTu8988 and SW1990 cells with Flag-MeCP2 induced EMT, as defined by a decrease in E-cadherin, ZO-1 and increases in vimentin, Snail, and N-cadherin. β-Tubulin was used as a loading control. **c** Knockdown of MeCP2 downregulated the active TGF-β1, p-Smad2/3. **d** MeCP2 overexpression resulted in upregulating the active TGF-β1, p-Smad2/3. **e**, **f** MeCP2 has no effects on TGF-β1R.

A previous study showed that EMT is associated with migration and invasion in cancer cells^14^. Therefore, we detected EMT markers at the protein level using western blotting. Our results revealed that MeCP2 knockdown induced upregulation of E-cadherin and ZO-1 and downregulation of vimentin, N-cadherin, and Snail (Fig. 4a). Conversely, MeCP2 overexpression resulted in downregulation of E-cadherin and ZO-1 and upregulation of vimentin, N-cadherin, and Snail (Fig. 4b). In addition, we found that the cells acquired a spindle-shaped morphology characteristic of EMT^15^ after overexpression of MeCP2 in PANC1, PaTu8988, and SW1990 cells (Supplementary Fig. S3a). Using immunofluorescence studies, we found that MeCP2 could promote vimentin and N-cadherin while inhibiting E-cadherin (Supplementary Fig. S4a–f). To further examine the mechanism by which MeCP2 affects EMT in pancreatic cancer cells, we investigated the effects of MeCP2 on classic TGF-β1/Smad signaling. The results showed that MeCP2 knockdown downregulated active TGF-β1 and p-Smad2/3 (Fig. 4c), while MeCP2 overexpression resulted in the upregulation of active TGF-β1 and p-Smad2/3 (Fig. 4d). MeCP2 knockdown or overexpression had no influence on total Smad2/3, total TGF-β1, or TGF-βR expression (Fig. 4c–f). In addition, we found that MeCP2 stimulated the activation of TGF-β1 in the supernatant (Supplementary Fig. S5a, b) and hardly affected the expression of TGF-β2 and TGF-β3 (Supplementary Fig. S5c, d). All these findings indicate that MeCP2 drives active TGF-β1/Smad signaling to promote EMT in pancreatic cancer cells.

### Exogenous TGF-β1 enhances migration, invasion, proliferation, and Smad2/3 phosphorylation in pancreatic cancer cells

**Fig. 5:**
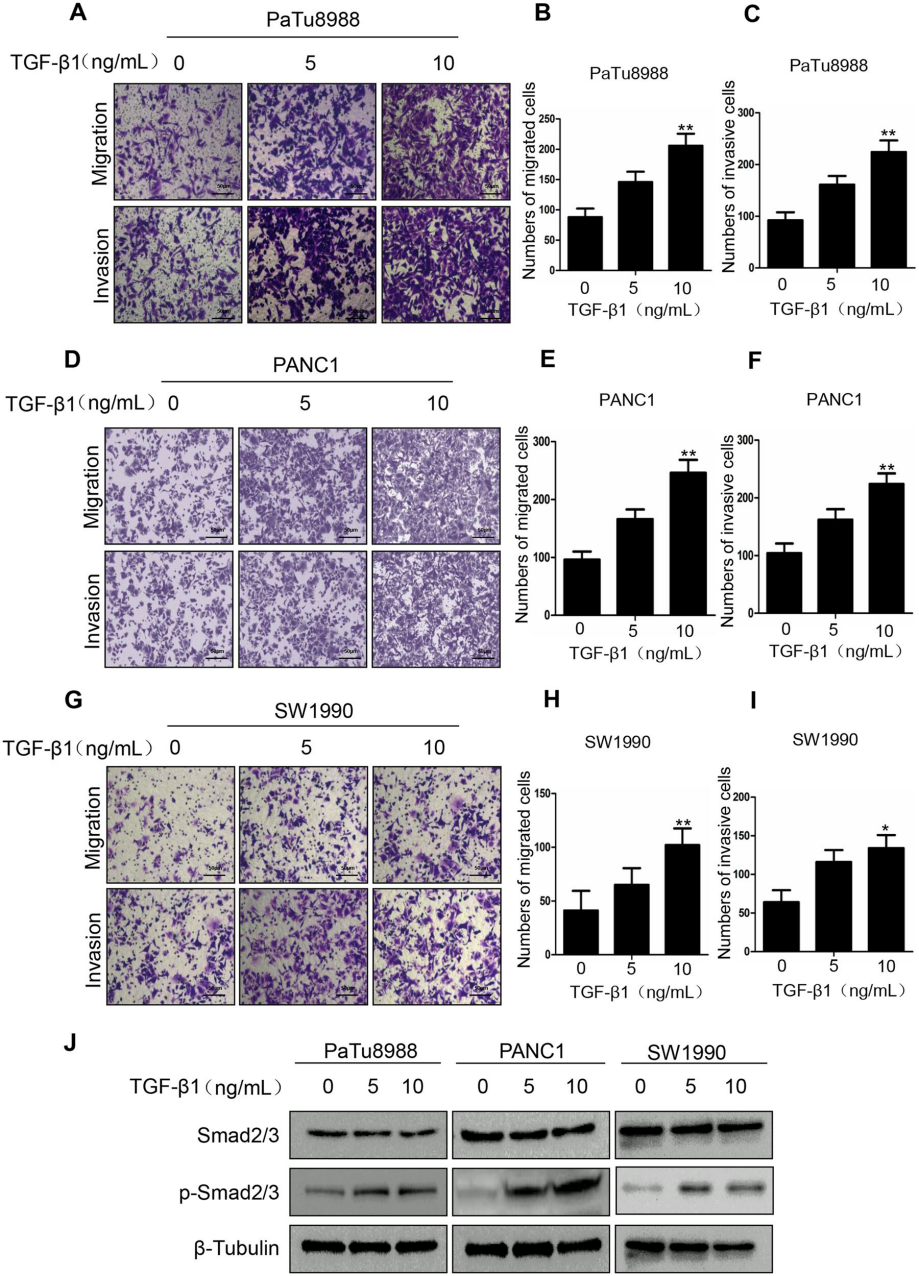
Exogenous TGF-β1 enhances the abilities of migration and invasion, and Smad2/3 phosphorylation. **a**–**g** The abilities of migration and invasion were examined in SW1990, PANC1, and PaTu8988 cells transfected with exogenous TGF-β1. Representative images of migrated cells are shown. The graph indicates the average number of migrated cells per field. *P < 0.05, **P < 0.01. **j** Exogenous TGF-β1 promotes p-Smad2/3.

To confirm whether pancreatic cancer cells are TGF-β1 responsive, we determined the effects of exogenous TGF-β1 on migration, invasion, and Smad2/3 phosphorylation in pancreatic cancer cells. We found that exogenous TGF-β1 significantly promoted the migration and invasion abilities of PaTu8988, PANC1, and SW1990 cells (Fig. 5a–i). In addition, we found that exogenous TGF-β1 promoted the growth of PaTu8988 and SW1990 cells (Supplementary Fig. S3b, c). Moreover, exogenous TGF-β1 enhanced Smad2/3 phosphorylation in these three cell lines (Fig. 5j). All these findings suggest that exogenous TGF-β1 can mimic the effect of MeCP2 overexpression on migration, invasion, and Smad phosphorylation in the three cell lines.

### MeCP2 regulates the expression of Furin to activate TGF-β1

**Fig. 6:**
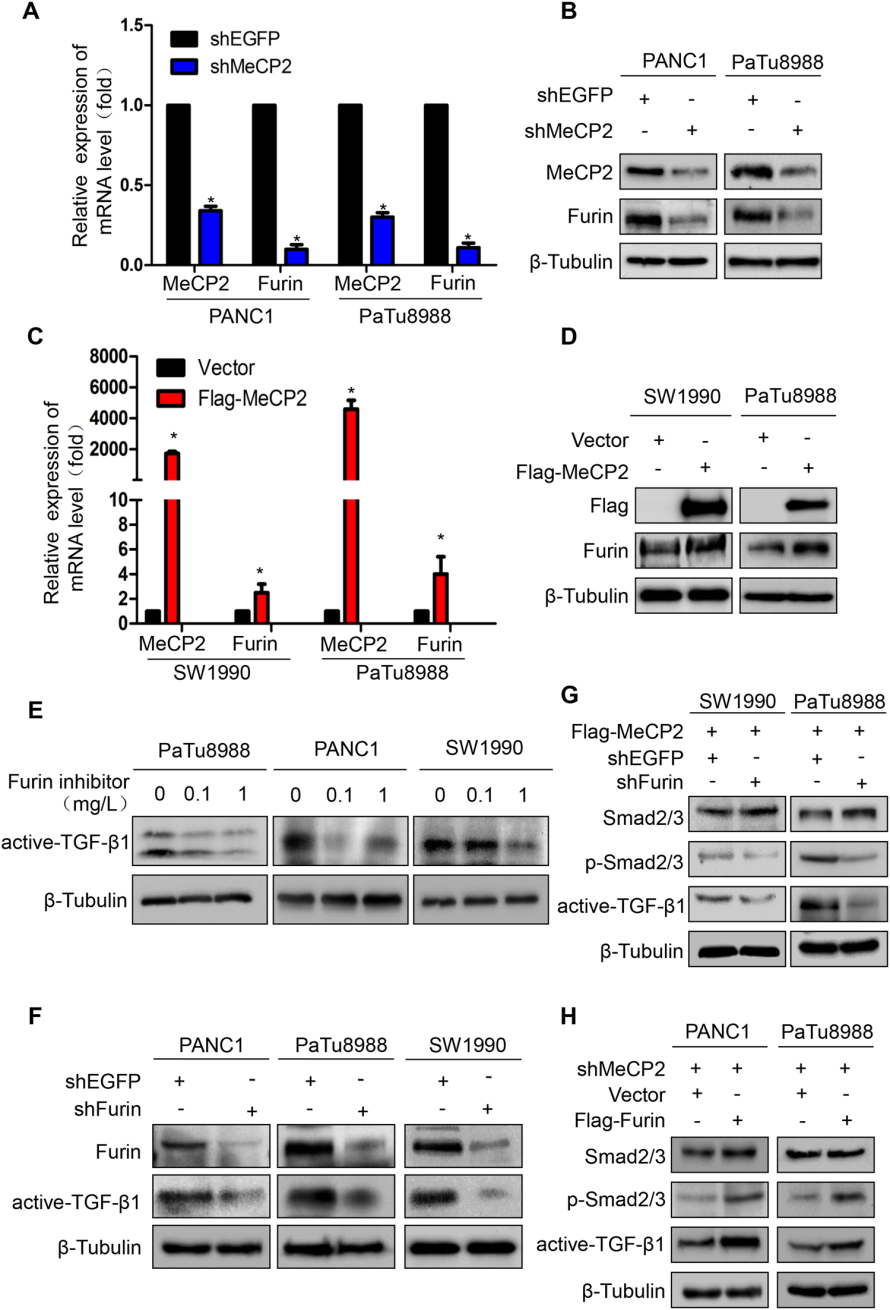
Methyl-CpG-binding protein 2 (MeCP2) regulates the expression of Furin to promote active TGF-β1. **a**, **b** The expression of Furin was examined using western blotting and real-time PCR in PANC1 and PaTu8988 cells transfected with shMeCP2. *P < 0.05. **c**, **d** The expression of Furin was examined using western blotting and real-time PCR in SW1990 and PaTu8988 cells transfected with Flag-MeCP2. *P < 0.05. **e** Pancreatic cancer cells were treated with Furin inhibitor at 0, 0.1, 1 µmol/L for 2 days. Active TGF-β1 expression was determined by western blotting. **f** Western blotting assays were performed to detect active TGF-β1 expression in pancreatic cancer cells treated with shFurin. **g** Western blotting assays were performed to confirm that knockdown of Furin can blunt the MeCP2 effect. **h** Western blotting assays were performed to confirm that Furin expression can rescue the MeCP2 effect.

TGF-β1 is deemed to be processed by the subtilisin-like proprotein convertase, Furin^5^. In this study, we found that exogenous TGF-β1 could induce Furin expression at both the mRNA and protein levels (Supplementary Fig. S3d, e). To further confirm the role of MeCP2 in the regulation of Furin, we transfected PANC1 and PaTu8988 cells with shEGFP or shMeCP2, and SW1990 and PaTu8988 cells with vector or Flag-MeCP2. As expected, MeCP2 knockdown downregulated Furin expression at the mRNA and protein levels compared with shEGFP expression (Fig. 6a, b). Accordingly, MeCP2 overexpression resulted in upregulation of Furin expression at the mRNA and protein levels (Fig. 6c, d). Further results showed that Furin knockdown or inhibitor decreased the expression of active TGF-β1 (Fig. 6e, f). In addition, after we transfected PaTu8988 and SW1990 cells with Flag-MeCP2, Furin knockdown downregulated active TGF-β1 and p-Smad2/3 (Fig. 6g), indicating that knockdown of Furin could blunt the MeCP2 overexpression effects. Conversely, Furin overexpression upregulated active TGF-β1 and p-Smad2/3 after we infected PANC1 and PaTu8988 cells with shMeCP2 virus suspension (Fig. 6h), indicating that overexpression of Furin could rescue the MeCP2 knockdown effects. All these results suggest that MeCP2 plays an important role in regulating Furin expression to enhance active TGF-β1.

### MeCP2 binds to Smad3 to promote Furin transcription

**Fig. 7:**
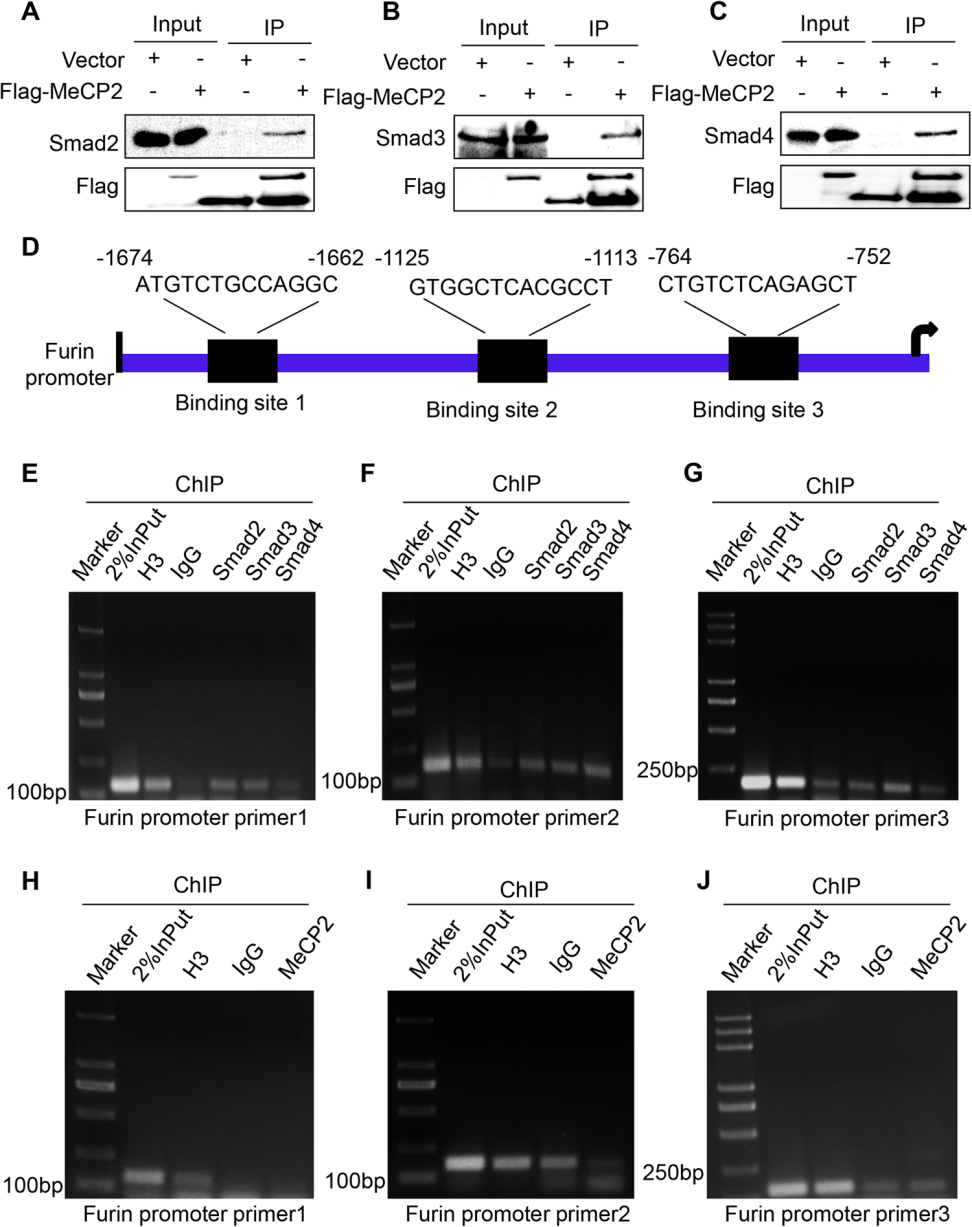
Methyl-CpG-binding protein 2 (MeCP2) binds to Smad3 to promote *furin* transcription. **a**–**c** Western blotting was used to analyze MeCP2 binding to the Smad2/3/4 in 293T cells via immunoprecipitation experiment. **d**–**j** Cross-linked chromatins from pancreatic cancer cells were incubated with antiserum against H3, IgG, Smad2, Smad3, and Smad4. DNA extracted from each immunoprecipitate was analyzed by standard PCR with three primers specific for *furin* promoter.

To characterize the interaction between MeCP2 and Smad2, Smad3, and Smad4, we first used immunoprecipitation assay to detect the interaction between MeCP2 and Smad2, Smad3, and Smad4. The results revealed that MeCP2 bound to Smad2, Smad3, and Smad4 (Fig. 7a–c). Then, we performed Chromatin immunoprecipitation (ChIP) assays to examine the binding ability of Smad2, Smad3, Smad4, and MeCP2 to three potential transcriptional binding sites on the *furin* promoter (Fig. 7d–j). Our data showed that Smad3 could bind to the promoter of three potential transcriptional binding sites of *furin* (Δ-1674-Δ-1662, Δ-1125-Δ-1113, and Δ-764-Δ-752), Smad2 could bind to site 1 (Δ-1674-Δ-1662), and site 2 (Δ-1125-Δ-1113) and Smad4 could only bind to site 2 (Fig. 7e–g), while MeCP2 could not bind to the promoter (Fig. 7h–j). Transcription factor-binding sites that are located closer to translational start sites are more relevant to gene transcriptional activity^16^. It has been suggested that Smad3 may have more influence on *furin* transcription than Smad2/4. In addition, we found that knockdown of MeCP2 could weaken the ability of Smad2/3/4 to bind to the *furin* promoter (Supplementary Fig. S5e–g). Thus, we proposed that Smad2/3/4, but mainly Smad3, bound to the *furin* promoter by interacting with MeCP2, to enhance the transcription of *furin*.

## Discussion

The above results indicate that MeCP2 may function as a promoter in pancreatic cancer. We confirmed that MeCP2 was upregulated in human pancreatic cancer and was directly related to clinicopathological features and stage. Furthermore, we found for the first time that the MeCP2-driven Smads–Furin-TGF-β1 axis represents a novel mechanism for promoting EMT in pancreatic cancer cells. All these findings suggest that MeCP2 may be a potential candidate for the diagnosis of pancreatic cancer.

Ever since the discovery that MeCP2 is an essential player in Rett syndrome (RTT), there has been considerable interest in obtaining a comprehensive understanding of this protein. However, the involvement of MeCP2 in pathologies other than RTT, such as tumorigenesis, remains poorly explored and understood. MeCP2 is upregulated in gastric, breast, colon, and prostate cancer^9^. In gastric cancer cells, MeCP2 was found to promote proliferation by activation of the MEK1/2–ERK1/2 signaling pathway through upregulating GIT112. Yadav et al.^17^ identified MeCP2 gene polymorphisms as candidates for breast cancer susceptibility, while Kedarlal Sharma et al.^18^ proved that MeCP2 overexpression inhibited the proliferation, migration, and invasion of C6 glioma cells. Nevertheless, to our knowledge, few studies have described the relationship between MeCP2 and EMT in pancreatic cancer cells.

It is well-known that EMT plays an important role in pancreatic carcinoma progression^19^. In this study, we report that MeCP2 promotes EMT by driving Furin/TGF-β1/Smad signaling in pancreatic cancer cells. TGF-β1 signaling is associated with the regulation of malignancy initiation, progression, and metastasis in mammary carcinoma, pancreatic cancer, glioblastoma, prostate carcinoma, and hepatocellular carcinoma^20^. When TGF-β1 is activated, Smad2 and Smad3 are phosphorylated and undergo dimerization with Smad4, thus allowing its translocation into the nucleus^21^. As expected, MeCP2 knockdown downregulated active TGF-β1 and p-Smad2/3, while MeCP2 overexpression upregulated active TGF-β1, and then activated p-Smad2/3, suggesting that MeCP2 activates TGF-β1/Smad signaling to regulate EMT.

The classical role of MeCP2 is in gene suppression through recruitment of histone deacetylases and co-repressor complexes to methylated CpG-sites^9^, which has been observed in numerous diseases, including Rett syndrome^22^, scleroderma fibroblasts^23^ and systemic sclerosis^24^. However, in recent studies, MeCP2 has been found to play an important role in transcription activation^25^. After studying thousands of genes, the majority of genes (~85%), such as Sst, Oprk1, Gamt, and Gprin117, appear to be activated by MeCP2^25^. Moreover, Furin is an authentic TGF-β1-converting enzyme and thus increases activated TGF-β1 levels^25^. In this study, we found a positive correlation between MeCP2 and Furin expression and confirmed that MeCP2 enhances Smad2/3/4, especially Smad3 binding to the *furin* promoter. Notably, our results establish a direct link between MeCP2, Smad3, and Furin in controlling EMT in pancreatic cancer cells.

In summary, our study provides the first evidence that MeCP2, Smad3, and Furin form positive feedback to promote EMT in pancreatic cancer cells. All these findings suggest that MeCP2 is a potential biomarker for pancreatic cancer therapy.

## Materials and methods

### Analysis of MeCP2 mRNA expression in human pancreatic cancer tissues

Correlations between pancreatic cancer histology and MeCP2 gene expression were determined through analysis of Badea and TCGA databases, which are available through Oncomine (Compendia Biosciences, www.oncomine.org) and UCSC (https://genome-cancer.ucsc.edu).

### Immunohistochemical staining

Seventy-eight pancreatic cancer tissues from Shanghai Outdo Biotech Company (NO.XT15-051) which were got patient informed consent and approved by the China Association For Ethical Studies were enrolled in this study. In this study, immunohistochemistry (IHC) was performed on tissue-microarrays. IHC was done following the standard protocol of Peroxidase Conjugated Mouse/Rabbit IgG SABC Kit (Catalog No: SA1020, Boster Biological Technology). The primary antibody was a rabbit monoclonal antibody against MeCP2 (Cell Signaling, CAT 3456, 1:500), and the secondary antibody was biotinylated goat anti-mouse/anti-rabbit IgG (Boster Biological technology, BA1056, 1:200). The chromogenic reaction was carried out with a DAB chromogenic kit (Catalog No: AR1022, Boster Biological technology) for 5 min, resulting in the expected brown-colored signal. Finally, after rinsing with deionized water, the slides were counterstained with hematoxylin, dehydrated, and mounted with mounting medium (Product No: D0547, Boster Biological technology) and coverslip. The colored cells are positive cells.

### Cell culture

The human pancreatic cancer cell lines (PANC1, PaTu8988 and SW1990) were bought from Shanghai Institutes for Biological Sciences (CAS), and the human embryonic kidney cell line (293T) was obtained from the American Type Culture Collection. All cell lines have been tested and authenticated by STR karyotype analysis. The cell lines were cultured with DMEM (Hyclone, China) supplemented with 10% fetal bovine serum (Gibco, USA) in humidified 5% CO_2_ incubator at 37 °C.

### Plasmid construction

The entire MeCP2 sequence was amplified with RT-PCR using primers MeCP2-all (Table 1), and then cloned into the expression vector p3xFLAG-Myc-CMV™-24 (Sigma, E9283). The MeCP2, EGFP shRNA oligos (Table 1) were firstly annealed into double-strands and then cloned into pLKO.1-puro (Sigma, SHC005).

**Table 1:** DNA and RNA nucleotide sequences.

MeCP2-F	CAGCGTCTGCAAAGAGGAGA
MeCP2-R	GCTCCTCTCTGTTTGGCCTT
MMP2-F	CACAGGAGGAGAAGGCTGTG
MMP2-R	GAGCTTGGGAAAGCCAGGAT
MMP9-F	TTCAGGGAGACGCCCATTTC
MMP9-R	TGTAGAGTCTCTCGCTGGGG
Furin-F	CCAAAGACATCGGGAAACG
Furin-R	TTAAACCCATCTGCGGAGTAG
GAPDH-F	TGGGGAAGGTGAAGGTCGG
GAPDH-R	CTGGAAGATGGTGATGGGA
TGFβR1-F	CTGTGAAGCCTTGAGAGTAA
TGFβR1-R	TGACTGAGTTGCGATAATGT
TGFβ2-F	GTGAAGAACTAGAAGCAAGA
TGFβ2-R	GCAATAACATTAGCAGGAGA
TGFβ3-F	TCAAGAAGCAGAAGGATCAC
TGFβ3-R	TGTCGGAAGTCAATGTAGAG
ChIP-PCR1-F	ACTAAACGGCCCATTGTTGTG
ChIP-PCR1-R	ACGTCACAGGATGGTGGTTAG
ChIP-PCR2-F	ATCCGACCCAAGATGTTGATAATG
ChIP-PCR2-R	CTGGTCTCGCACTCCTGAC
ChIP-PCR3-F	GTGGTCTCTGGCTTCCTATGGAC
ChIP-PCR3-R	CTTCCGCCAGCTCCAGCTC
MeCP2-all-F	CCGGAATTCAATGGTAGCTGGGATGTTAGG
MeCP2-all-R	CGGGGTACCGCTAACTCTCTCGGTCACGG
shMeCP2-F	CCGGGAGAGCGCAAAGACATTGTTTCTCGAGAAACAATGTCTTTGCGCTCTCTTTTTG
shMeCP2-R	AATTCAAAAAGAGAGCGCAAAGACATTGTTTCTCGAGAAACAATGTCTTTGCGCTCTCG
shEGFP-F	CCGGTACAACAGCCACAACGTCTATCTCGAGATAGACGTTGTGGCTGTTGTATTTTTG
shEGFP-R	AATTCAAAAATACAACAGCCACAACGTCTATCTCGAGATAGACGTTGTGGCTGTTGTA

### Transfection of the plasmid, viral infection, and Furin inhibitors

In total, 5 × 10^5^ PaTu8988 and SW1990 cells were seeded in a six-well plate per well for 12 h followed by transfection with a vector or Flag-MeCP2 via Lipofectamine 2000 reagent (Invitrogen, Carlsbad, CA, USA), in the light of the manufacturer’s instructions. In HEK293T cells, the plasmid shEGFP or sh- MeCP2 was co-transfected with psPAX2 and pMD2.G, and the supernatants were harvested and concentrated following 48 h and 72 h. PANC1 and PaTu8988 cells were treated with 1 × 10^6^ virus suspension of either shEGFP or shMeCP2 in 8 mg/ml polybrene (Sigma-Aldrich) existence for 2 days. Cells were then cultivated with puromycin (1:10,000 dilution), while the cells in a blank group were all unviable. Cells were incubated in culture medium with 10% FBS supplemented with Furin inhibitors Hexa-D-arginine (MCE, HY-P1028) over the concentration range 0, 0.1, and 1 mg/L. Cells were harvested at 48 h after treatment.

### Western blotting

The cultured cells were rinsed with cold PBS before treated with cell lysis buffer aids (2 × loading buffer, 2 µg/ml aprotinin, 1 mM PMSF, 2 mM β-mercaptoethanol) at 100 °C for 10 min. Then the mixture was centrifuged under 4 °C at 12000 rpm for 10 min. About 25–30 μg of protein was loaded in each lane, separated by 10% SDS-PAGE, and transferred to the PVDF membrane. The membrane was blocked with 5% nonfat milk powder for 1 h at room temperature before overnight incubation with primary antibodies at 4 °C, followed by the secondary antibody. The blots were incubated with primary antibodies rabbit anti-MeCP2 (Cell Signaling, CAT 3456), rabbit anti-Furin (Proteintech, CAT 18413-1-AP), anti-TGF-β1 (Cell Signaling, CAT 3709), anti-TGF-βR (Cell Signaling, CAT 3712), anti-MMP2 (Immunoway, CAT YT2798), anti-MMP9 (Immunoway, CAT YT1892), EMT Kit (Cell Signaling, CAT 9782), Smad2/3 Antibody Sampler Kit (Cell Signaling, CAT 12747), mouse anti-Flag (SIGMA, CAT 6631), mouse anti-β-Tubulin (Cell Signaling, CAT4466).

### Cell proliferation assay

Cell proliferation was measured by Cell Counting Kit-8 (CCK8). Forty-eight hours later after transfection, cell suspension containing 1000–1500 cells (100 μL) was seeded into each well of a 96-well culture plate and continued to incubate at 37 °C. At 1, 2, 3, 4, and 5 days, 100 μl serum-free culture medium and 10 μl CCK8 solutions were added to each well, followed by incubation at 37 °C for 2 h. The absorbance was measured with a plate reader at 450 nm on an ELX-800 spectrometer reader. Six independent samples were detected in each experimental group. Cell proliferation rate = (OD_experimental_ − OD_blank_)/(OD_control_ − OD_blank_) × 100%.

### Xenograft mouse model

The protocol was approved by the Institutional Animal Care and Use Committee of Jiangsu University, Zhenjiang, China. PANC1 and SW1990 cells (2.0 × 10^6^ cells/site) stably transfected with shEGFP, shMeCP2, vector, and Flag-MeCP2 were subcutaneously injected into 5-week-old BALB/c nude mice (Shanghai SLAC Laboratory Animal Co., Ltd, Shanghai, China) to generate xenografts. Five female mice were randomly distributed to each group. There is no blinding. The tumors were collected from the fourth week. The tumor volume was measured every week after injection and calculated using the following formula: length × (width^2^)/2.

### Transwell and invasion assay

Transwell and invasion assays were carried out using matrigel chambers (BD Biosciences) according to the manufacturer’s protocol. Transfected PANC1, PaTu8988, and SW1990 cells were harvested and resuspended in serum-free medium, cell suspensions containing 50,000 cells (100 μL) were plated into the upper chamber of the transwell, and DMEM (500 μl) containing 10% FBS was added to the lower chamber of the transwell. The system was incubated at 37 °C, 5% CO_2_ atmosphere for 24 h. The cells on the upper surface were scraped and washed away, whereas the migrated and invaded cells on the lower surface were fixed and stained with 0.05% crystal violet for 30 min. Finally, migrated and invaded cells were counted, and the relative number was calculated by software Image J.

### Real-time PCR

The total RNA was isolated using RNAiso Plus (Takara). Reverse transcription was performed using RevertAid First Strand cDNA Synthesis Kit (Thermo) according to the manufacturer’s recommendations. Real-time PCR was performed in triplicate in 20-μl reactions with iQ SYBR^®^ Premix Ex Taq™ Perfect Real Time (Bio-Rad Laboratories, Inc.), 50 ng of first-strand cDNA and 0.2 μg each primer. Samples were cycled once at 95 °C for 2 min, and then subjected to 35 cycles of 95, 56, and 72 °C for 30 s each. The relative mRNA content was calculated using the 2^−ΔΔCT^ method with GAPDH as an endogenous control. Primers are listed in Table 1.

### Immunofluorescence

Cells were seeded on glass coverslips in 24-well plates and grew to subconfluence, then infected with shEGFP, shMeCP2, vector, or Flag-MeCP2 for 48 h. Routinely, cells were washed with PBS, fixed with ice-cold 100% methanol for 15 min at room temperature, and blocked with 3% BSA in PBS for 1 h. Cells were incubated with primary antibody overnight at 4 °C, and then incubated with secondary antibodies for 1 h at room temperature in the dark, DAPI (1 μg/ml, Pierce, IL, USA) counterstain was used for nuclear staining. After extensive washing, the coverslips were then mounted on glass slides, and the fluorescent images were captured with a fluorescent microscope and a SPOT CCD camera.

### Immunoprecipitation (IP)

293T cells were lysed in co-IP buffer (10 mM HEPES [pH 8.0], 300 mM NaCl, 0.1 mM EDTA, 20% glycerol, 0.2% NP-40, protease, and phosphatase inhibitors). Lysates were centrifuged and cleared by incubation with 25 μl of Protein A/G beads (Thermo Fisher Scientific, MA, USA) for 1 h at 4 °C. The pre-cleared supernatant was subjected to IP using the indicated first antibodies at 4 °C overnight. Then, the protein complexes were collected by incubation with 30 μl of protein A/G beads for 3–4 h at 4 °C. The collected protein complexes were washed with co-IP buffer and analyzed by western blotting.

### ChIP analysis

ChIP was performed using Simple ChIP Plus Enzymatic chromatin IP Kit (Agarose Beads) (Cell Signaling, CAT9004) according to the manufacturer’s recommendations. Cells were fixed with formaldehyde and lysed, and then chromatin was fragmented by partial digestion with micrococcal nuclease automatic fragmentation to obtain chromatin fragments of 1–6 nucleosomes. Chromatin immunoprecipitations were performed using 1–2 μl of input, IgG, H3, Smad2, Smad3, Smad4 antibodies, and ChIP-Grade Protein G Agarose Beads. After reversal of protein–DNA cross-links, the DNA was purified using DNA purification spin columns.

### ChIP-PCR

According to the online primer program (http://jaspar.genereg.net/), three potential transcriptional binding sites were predicted. Primers are listed in Table 1. The enrichment of particular DNA sequences of PaTu8988 cells was analyzed by semi-quantitative PCR. Semi-quantitative PCR products were separated on 1.5% agarose gels, and visualized under UV illumination. The reaction mixture contains 2 × green PCR mix 10 µl, 5 µM primers 1 µl, nuclease-free water 7 µl, and the appropriate DNA sample 2 µl. The 2% input chromatin DNA was diluted five times. Reactions were carried out in a thermal cycler under the following conditions: 95 °C for 5 min followed by 95 °C for 30 s, 58 °C for 30 s, 72 °C for 30 s for 35 cycles, 72 °C for 5 min.

### Data analysis

All data are presented as mean ± s.d. from at least three independent experiments. Comparisons between groups were analyzed using the Student’s *t* test (two groups) or the one-way ANOVA (multiple groups). *P* < 0.05 was considered statistically significant.

## Supplementary information

Figure S1

Figure S2

Figure S3

Figure S4

Figure S5

supplementary legends

Table 1
