# Supplementary figures and images for: Methyl-CpG-binding protein 2 drives the Furin/TGF-β1/Smad axis to promote epithelial–mesenchymal transition in pancreatic cancer cells
Source: Oncogenesis. 2020 Aug 26;9(8):76. doi: 10.1038/s41389-020-00258-y (PMC7450052; doi:10.1038/s41389-020-00258-y)

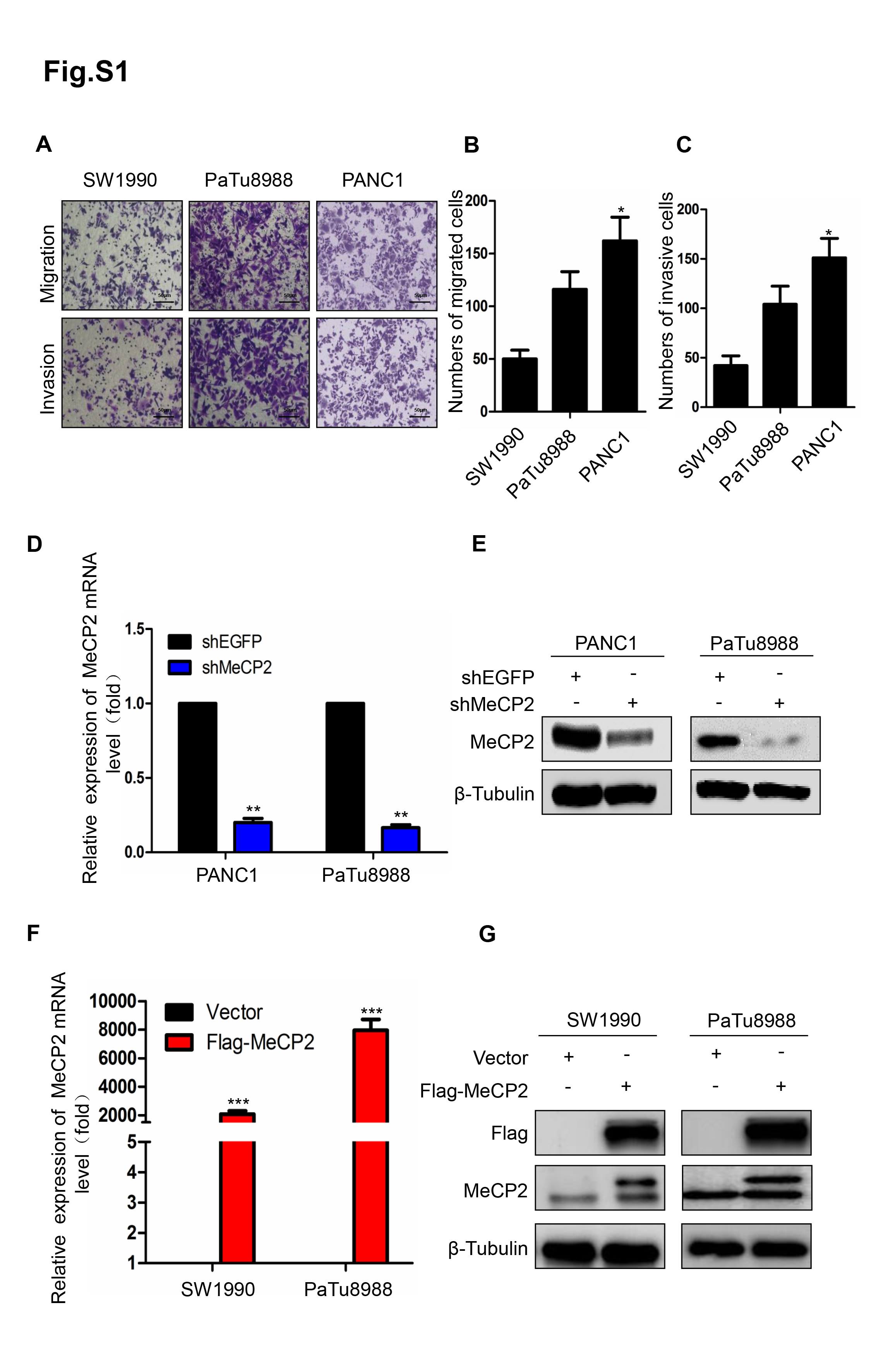

Supplement: Supplementary file 1 — Figure S1 [file 41389_2020_258_MOESM1_ESM.tif]

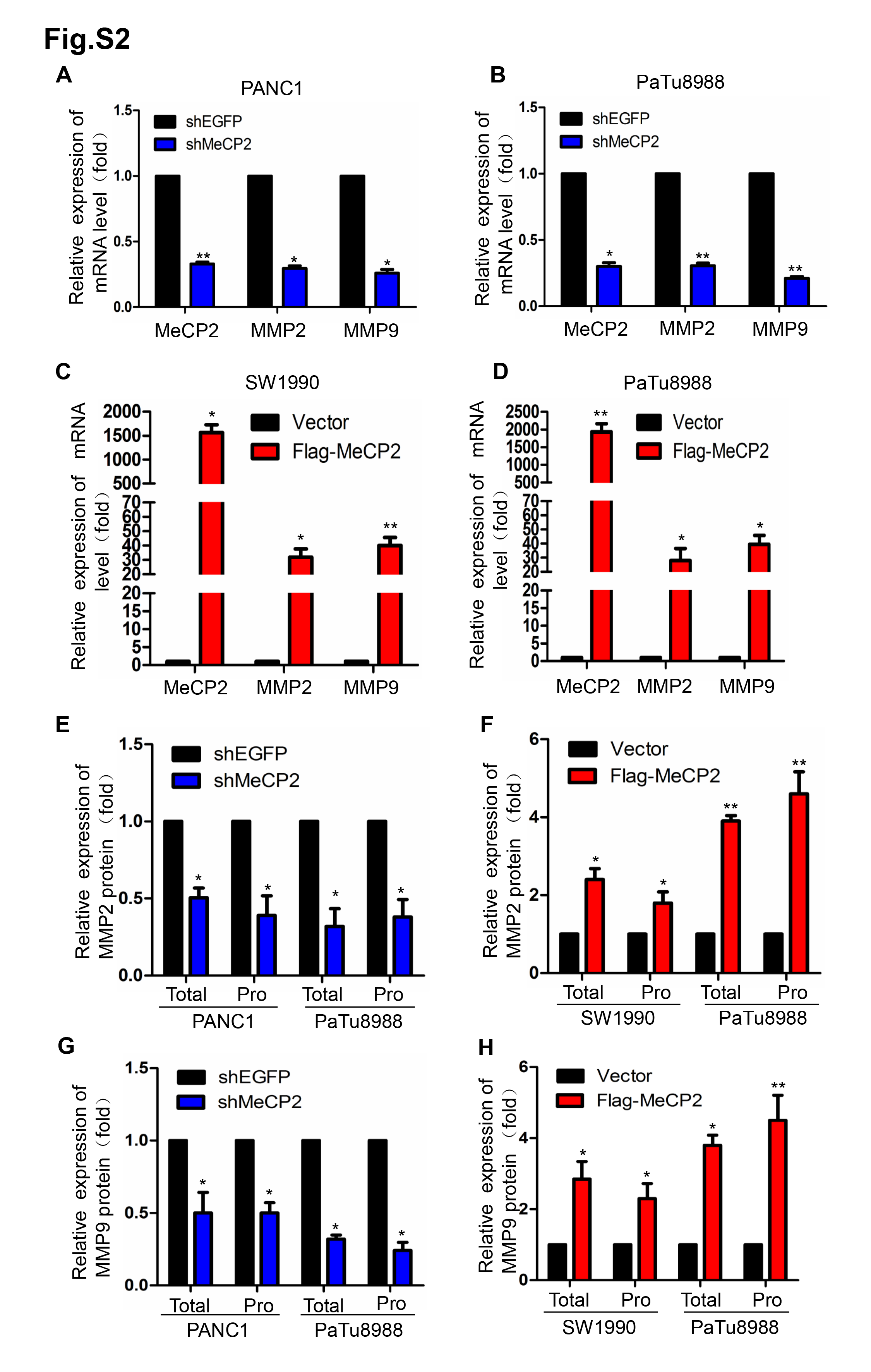

Supplement: Supplementary file 2 — Figure S2 [file 41389_2020_258_MOESM2_ESM.tif]

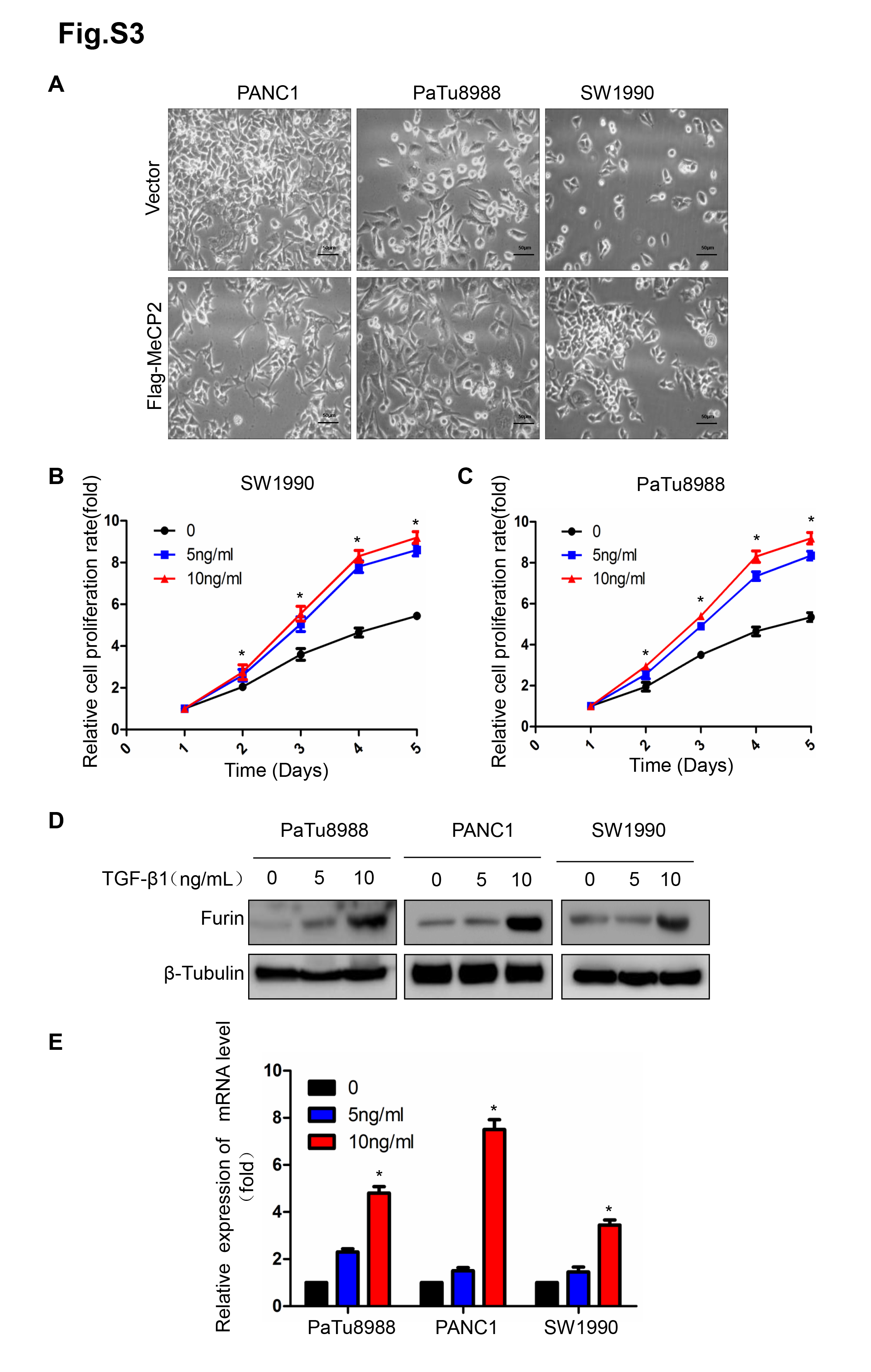

Supplement: Supplementary file 3 — Figure S3 [file 41389_2020_258_MOESM3_ESM.tif]

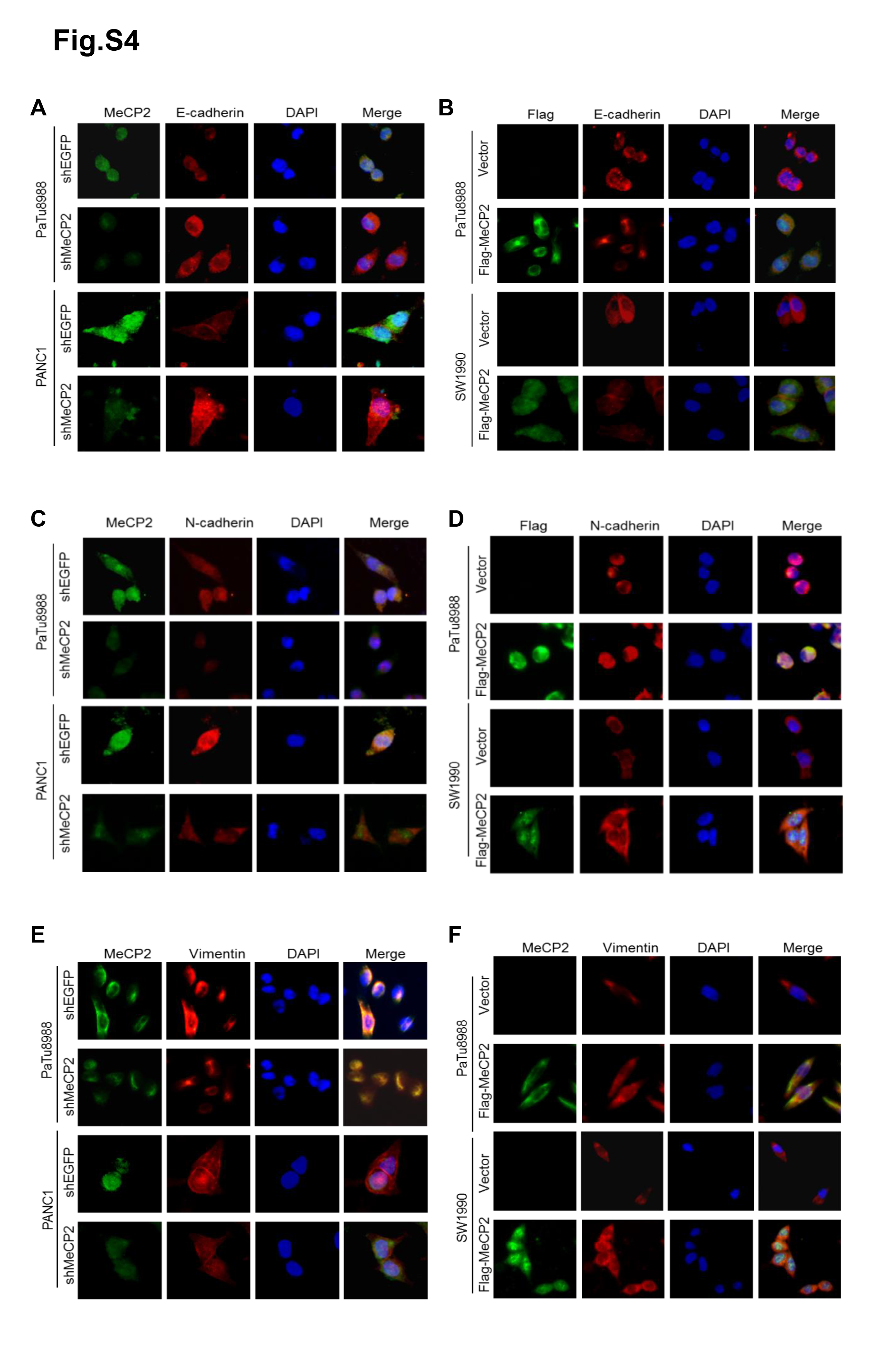

Supplement: Supplementary file 4 — Figure S4 [file 41389_2020_258_MOESM4_ESM.tif]

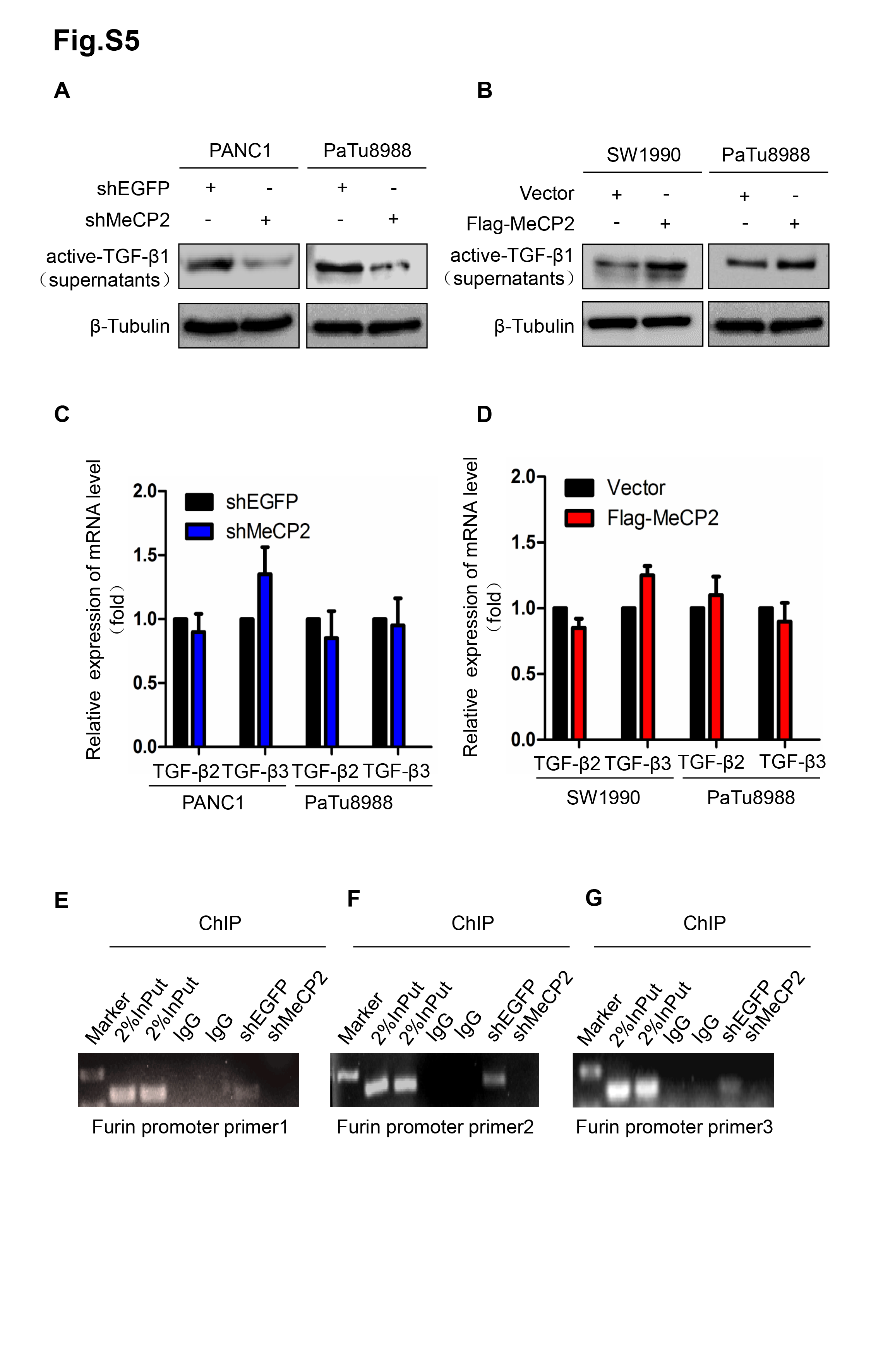

Supplement: Supplementary file 5 — Figure S5 [file 41389_2020_258_MOESM5_ESM.tif]
