# Supplementary material for: Methyl-CpG-binding protein 2 drives the Furin/TGF-β1/Smad axis to promote epithelial–mesenchymal transition in pancreatic cancer cells
Source: Oncogenesis. 2020 Aug 26;9(8):76. doi: 10.1038/s41389-020-00258-y (PMC7450052; doi:10.1038/s41389-020-00258-y)
Supplement: Supplementary file 6 — supplementary legends [file 41389_2020_258_MOESM6_ESM.docx]

**Fig.S1**. (A, B, C) The abilities of migration and invasion in PANC1, PaTu8988, SW1990 cells were detected. **P*<0.05. (D, E) MeCP2 protein and mRNA levels were reduced in shMeCP2-PANC1 and PaTu8988 cells. ***P*<0.01. (F, G) MeCP2 protein and mRNA levels were increased in PaTu8988 and SW1990 cells transfected with Flag-MeCP2. ****P*<0.001.

**Fig.S2**. (A, B) MMP2 and MMP9 mRNA levels were reduced in shMeCP2-PANC1 and PaTu8988 cells. **P*<0.05, ***P*<0.01. (C, D) MMP2 and MMP9 mRNA levels were increased in PaTu8988 and SW1990 cells transfected with Flag-MeCP2. **P*<0.05, ***P*<0.01. (E-H) The quantification of MMP2/MMP9 (pro- and totals) is shown. **P*<0.05, ***P*<0.01.

**Fig.S3**. (A) The cells acquired a spindle-shaped morphology characteristic of EMT in PANC1, PaTu8988 and SW1990 cells after overexpression of MeCP2. (B) At 1, 2, 3, 4 and 5 days after exogenous TGF-β1, SW1990 cell proliferation was determined by CCK8 assays. **P*<0.05. (C) At 1, 2, 3, 4 and 5 days after exogenous TGF-β1, PaTu8988 cell proliferation was determined by CCK8 assays. **P*<0.05. (D) Furin was identified using western blotting. (E) *furin* was identified using RT-PCR.

**Fig.S4**. (A-F) Using immunofluorescence studies, we found that MeCP2 could promote Vimentin and N-cadherin, while inhibit E-cadherin. Also, the cells acquired a spindle-shaped morphology characteristic of EMT after MeCP2 overexpression.

**Fig.S5**. (A, B) Active-TGF-β1 in the culture supernatants was detected by western bolt. (C, D) MeCP2 has no effect on TGF-β2 or TGF-β3 in pancreatic cancer cells. (E, F, G) Cross-linked chromatins from pancreatic cancer cells were incubated with antiserum against IgG, Smad2. DNA extracted from each immunopecipitate was analyzed by standard PCR with three primers specific for furin promoter.
